# Supplementary material for: Antimicrobial resistance and molecular typing of Staphylococcus aureus isolates from raw milk in Hunan Province
Source: PeerJ. 2023 Aug 28;11:e15847. doi: 10.7717/peerj.15847 (PMC10470458; doi:10.7717/peerj.15847)
Supplement: Supplemental Information 1 [file peerj-11-15847-s001.zip › gene sequencing/gmk/Multilocus sequence typing of Staphylococcus.pdf]

Multilocus sequence typing of *Staphylococcus aureus* isolates recovered from cows with mastitis in Brazilian dairy herds

Renata F. Rabello,[1] Beatriz M. Moreira,[1] Regina M. M. Lopes,[1] Lúcia M. Teixeira,[1] Lee W. Riley[2] and Angela C. D. Castro[1]

[1]Instituto de Microbiologia, Universidade Federal do Rio de Janeiro, Rio de Janeiro, Brazil

[2]School of Public Health, University of California, Berkeley, CA, USA

Correspondence
Angela C. D. Castro
acastro@micro.ufrj.br

Received 20 April 2007
Accepted 4 July 2007

Staphylococcus aureus is a major pathogen associated with bovine mastitis, one of the most important infectious diseases occurring in dairy cattle herds worldwide. In the present study, *S. aureus* isolates recovered from cows with mastitis in dairy herds located in the south-east of Brazil were genotyped by PFGE and multilocus sequence typing (MLST). PFGE identified 60 pulsotypes (PTs), which were found to be distributed among six clonal complexes (CCs) by MLST. All PTs with similarity percentages greater than 65 % belonged to the same CC. Most of the PTs belonged to CC126 ($n=28$) and CC97 ($n=19$), which were represented by 91 % of the isolates. These CCs have also been recovered from cows with mastitis in countries located in different continents, but they have rarely been isolated from human specimens. Few isolates were represented by PTs belonging to CCs that are frequently isolated from human specimens (CC1, CC5 and CC30). These data reinforce the hypothesis that a limited number of *S. aureus* CCs are responsible for most bovine mastitis cases internationally. Specific features of the specialized clones should be studied for use as future targets of mastitis control measures.

INTRODUCTION

Mastitis is one of the most important infectious diseases occurring in dairy cattle herds, causing significant financial losses worldwide. *Staphylococcus aureus* is a major pathogen associated with bovine mastitis (Wilson *et al.*, 1997; Brito *et al.*, 1999).

The results of different studies, with a few exceptions (Joo *et al.*, 2001), suggest that relatively few *S. aureus* clones are responsible for the majority of bovine intramammary infections and that these clones have a broad geographical distribution (Matthews *et al.*, 1994; Kapur *et al.*, 1995; Fitzgerald *et al.*, 1997; Mork *et al.*, 2005). Interestingly, the most prevalent bovine mastitis clones have rarely been recovered from humans, suggesting a host specificity of *S. aureus* clones (Kapur *et al.*, 1995; Larsen *et al.*, 2000). However, Mork *et al.* (2005) observed that common genotypes caused mastitis in cows, goats and sheep, suggesting anatomical site specificity rather than host specificity.

Most epidemiological studies have used techniques that rely on comparison of electrophoretic patterns, such as PFGE (Mork *et al.*, 2005), random amplified polymorphic DNA analysis (Matthews *et al.*, 1994; Fitzgerald *et al.*, 1997), ribotyping (Fitzgerald *et al.*, 1997; Larsen *et al.*, 2000) and multilocus enzyme electrophoresis (Kapur *et al.*, 1995; Fitzgerald *et al.*, 1997). Although most of these techniques are highly discriminatory, interlaboratory comparisons may be difficult. To overcome this disadvantage, more reproducible methods based on sequencing of gene fragments, such as multilocus sequence typing (MLST), have been developed. MLST is amenable to longer-term and global epidemiological studies as it is based on variation that accumulates relatively slowly. This method has a high discriminatory power and the data can be compared across laboratories by making them accessible through the Internet (Enright & Spratt, 1999).

MLST has been used to characterize and investigate the distribution of *S. aureus* clones associated with human infections (Enright *et al.*, 2000, 2002; Feil *et al.*, 2003; Robinson & Enright, 2004; Vivoni *et al.*, 2006) and with bovine mastitis (Kwon *et al.*, 2005; Smith *et al.*, 2005a, b). However, to date, MLST data on bovine isolates are still scarce. Additional studies are necessary to determine the distribution of the clones associated with bovine intramammary infections worldwide and to confirm whether a few specialized clones are responsible for the majority of mastitis cases. A better understanding of the epidemiology

Abbreviations: CC, clonal complex; MLST, multilocus sequence typing; MRSA, meticillin-resistant *Staphylococcus aureus*; PT, pulsotype; ST, sequence type.

of this pathogen may contribute to the development of more effective measures for the control of bovine mastitis.

The purpose of the present study was to investigate the genetic diversity of *S. aureus* isolates from cows with mastitis in dairy herds located in Brazil and to examine whether any of the Brazilian *S. aureus* isolates were related to internationally distributed lineages.

METHODS

Bacterial isolates and identification. A total of 227 *S. aureus* isolates was included in this investigation. They were recovered from milk samples collected from cows with subclinical mastitis (with the exception of 2 isolates from clinical mastitis) belonging to 18 dairy herds distributed among 9 municipal districts of Rio de Janeiro state (Fig. 1), located in the south-eastern region of Brazil, between July 2001 and July 2004. The distance between these municipal districts ranged from 31 to 269 km. Milk samples were collected, according to the National Mastitis Council (1999) recommendations, from individual mammary quarters with a suspicious or positive reaction in the California mastitis test (milk thickening or gel formation), and from two animals showing signs of mastitis (presence of clots or blood in milk, or mammary quarter inflammation). A 0.01 ml volume of each milk sample was inoculated onto sheep blood agar and incubated for 24–48 h at 37 °C. Bacterial strains were identified by colony morphology on sheep blood agar and by conventional phenotypic methods, including catalase, tube coagulase and Voges–Proskauer tests (National Mastitis Council, 1999). A PCR assay using primers specific for a *S. aureus* 16S rRNA gene fragment was used to confirm the bacterial species identification (Lovseth *et al.*, 2004).

PFGE. All bacterial isolates were analysed by PFGE as described previously (Rabello *et al.*, 2005). Chromosomal DNA was digested with *Sma*I (Roche). Fragments were separated in a 1.0 % agarose gel in a CHEF-DRIII system (Bio-Rad) with pulse times increasing from 2 to 35 s over 21 h at 13 °C at a voltage gradient of 6 V cm^{-1}. Analysis of *Sma*I restriction patterns was performed initially by visual inspection, as suggested by Tenover *et al.* (1995). Isolates with identical restriction patterns in terms of size and number of bands were assigned to the same pulsotype (PT). PTs that differed from the main PTs by one to three bands were considered to be closely related. Computer-assisted analysis was performed with the MOLECULAR ANALYST FINGERPRINTING PLUS software package, version 1.6, of the Image Analysis System (Bio-Rad). The Dice coefficient and a tolerance of 0.5 % were used to calculate the similarity matrix, and a dendrogram was generated by the unweighted pair group method with arithmetic mean.

MLST. A total of 62 *S. aureus* isolates, representing all PTs identified by PFGE in the present study, were analysed by MLST as described by Enright *et al.* (2000). PCR products were purified using a QIAquick PCR purification kit (Qiagen), as recommended by the manufacturer, and sequenced. LASERGENE 6.0 software (DNASTAR) was used to analyse the sequences. Alleles and sequence types (STs) were identified using the MLST database (http://www.mlst.net). Forward and reverse trace files of putative novel alleles and the allelic profiles of novel STs were sent to the database curator for allele or ST number assignment and entry into the database. Subsequently, the isolates were grouped in clonal complexes (CCs) and analysed in conjunction with the entire *S. aureus* MLST database (http://saureus.mlst.net) with the program eBURST v3 [enhanced version of BURST (Based Upon Related Sequence Types)] (Feil *et al.*, 2004).

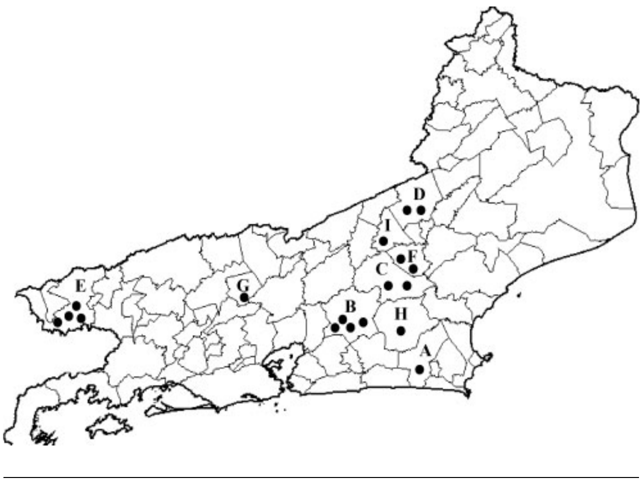

Fig. 1. Map of Rio de Janeiro state, Brazil, showing the distribution of the municipal districts and dairy herds from where the *S. aureus* isolates were recovered. Each capital letter and black circle corresponds to one municipal district and one herd investigated, respectively.

RESULTS AND DISCUSSION

The genetic diversity of the 227 *S. aureus* bovine isolates included in the present study was initially assessed by PFGE. Between 7 and 12 bands ranging from 45.8 to 436.5 kb were included in the analysis and yielded 60 PTs. A total of 13 PTs (PT7, PT8, PT11, PT12, PT19, PT23, PT31, PT33, PT41, PT42, PT48, PT50 and PT58) were common to multiple herds and were represented by 122 isolates (53.8 %). PT7, PT11, PT19 and PT31 were isolated from six or seven herds, which were distributed in four to seven distinct municipal districts. These four PTs accounted for 93 (41 %) of the 227 isolates (Fig. 2).

PT11 was the most frequently isolated PT, representing 20.3 % (n=46) of all isolates. Another 16 PTs (PT2, PT5–PT8, PT12–PT20, PT23 and PT24) differed by up to three bands from PT11 by visual inspection and were thus considered to be closely related to PT11. In general, these PTs were found in herds where PT11 was also present. These 17 PTs were represented by 113 isolates (49.8 %), which were recovered from 11 herds (Table 1, Fig. 2). In the dendrogram generated by computer-assisted analysis (Fig. 2), the similarity among these PTs ranged from 74 to 96 %. Likewise, another six PTs (PT30, PT32–PT36) were considered to be closely related to PT31 as they had up to three band differences when compared with PT31. Except for PT33, these were recovered only from herds where PT31 was also isolated. Approximately 11 % (n=24) of all isolates belonged to one of these seven PTs with similarity percentages ranging from 80 to 96 % (Table 1, Fig. 2).

Different PTs, ranging from 2 to 13 PTs, were also observed within almost all herds. However, closely related PTs were observed within several of these herds, suggesting that they may have a common ancestor (Table 1).

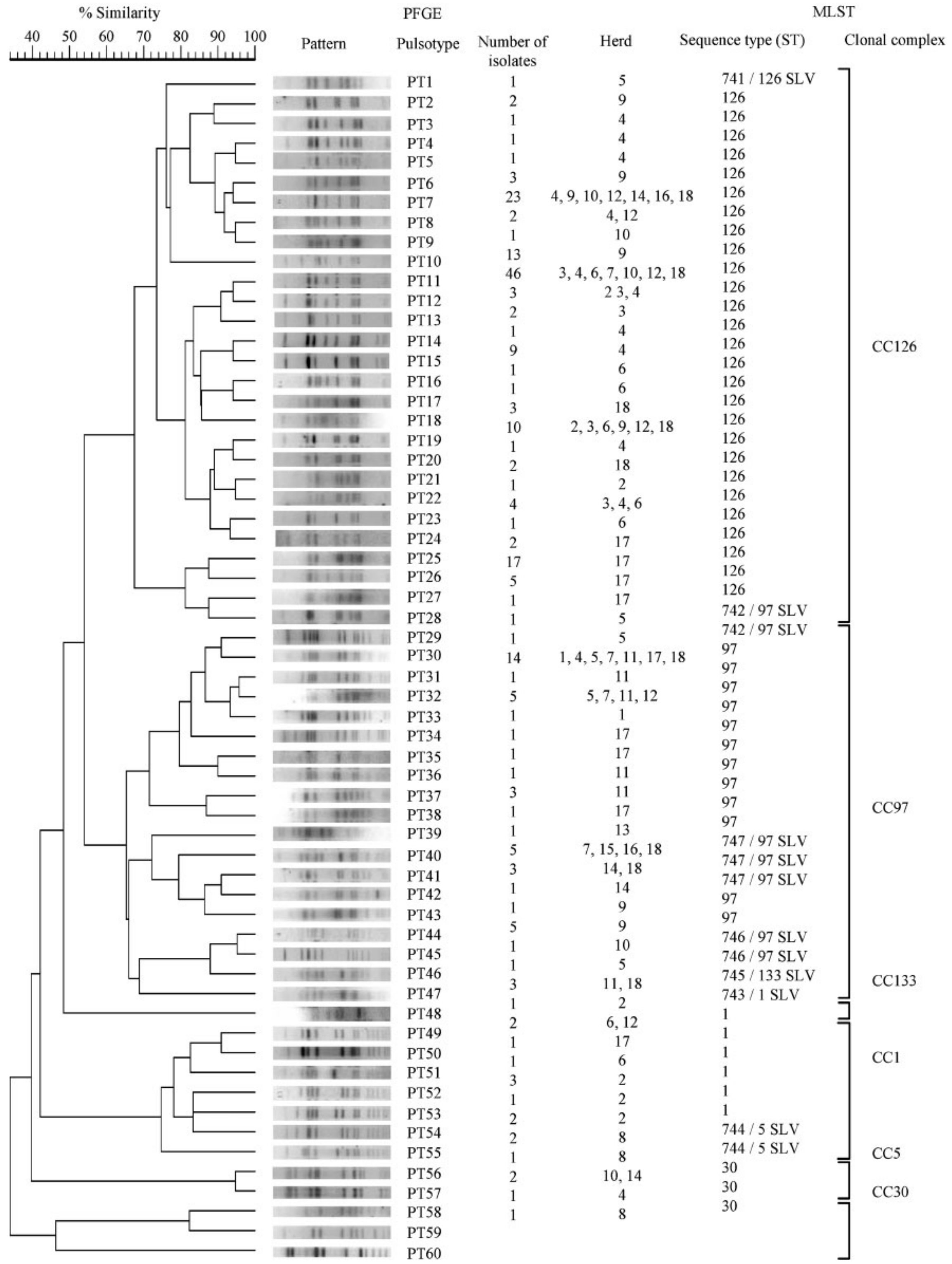

Fig. 2. Dendrogram of the PFGE patterns observed among *S. aureus* isolates recovered from cows with bovine mastitis in herds located in Rio de Janeiro state, Brazil. The STs related to each pattern and the CCs to which these STs belong are also shown.

Table 1. Distribution of PFGE PTs of *S. aureus* isolates recovered from cows with mastitis belonging to dairy herds from Rio de Janeiro state, Brazil

Herd	Municipal district	Year*	No. of isolates	PFGE PT (no. of isolates)†	CC‡
1	A	2002	2	[PT31 (1), PT34 (1)]	CC97
2	B	2001	9	[PT19 (1), PT22 (1)], [PT49 (1), **PT53 (3)**], PT54 (1), PT55 (2)	CC1, CC126
3	B	2001	12	[**PT11 (7)**, PT12 (1), PT13 (2), PT19 (1), PT23 (1)]	CC126
4	B	2001	24	[PT3 (1), PT4 (1)], [PT5 (1), PT7 (1), PT8 (1)], [PT11 (1), PT12 (2), PT14 (1), **PT15 (9)**, PT20 (1), PT23 (2)], PT31 (2), PT59 (1)	CC30, CC97, CC126
5	B	2002	6	PT1 (1), PT29 (1), [PT30 (1), PT31 (1), PT33 (1)], PT47 (1)	CC97, CC126
6	C	2002	19	[**PT11 (10)**, PT16 (1), PT17 (1), PT19 (3), PT23 (1), PT24 (1)], PT50 (1), PT52 (1)	CC1, CC126
7	C	2002	4	PT11 (1), [PT31 (1), PT33 (1)], PT41 (1)	CC97, CC126
8	D	2001	4	[PT56 (2), PT57 (1)], PT60 (1)	CC5, CC30
9	D	2001	27	[PT2 (2), **PT6 (3)**, PT19 (1)], [PT7 (2), **PT10 (13)**], [PT44 (1), **PT45 (5)**]	CC97, CC126
10	E	2003	10	[PT7 (2), **PT11 (5)**], PT9 (1), PT46 (1), PT58 (1)	CC30, CC97, CC126
11	E	2003	10	PT31 (2), [PT32 (1), PT33 (2), **PT38 (3)**], PT37 (1), PT48 (1)	CC97, CC133
12	E	2004	11	[**PT7 (6)**, PT8 (1), PT11 (1), PT19 (1)], PT33 (1), PT50 (1)	CC97, CC126
13	E	2004	1	PT40 (1)	CC97
14	F	2004	4	PT7 (1), [PT42 (1), PT43 (1)], PT58 (1)	CC30, CC97, CC126
15	F	2004	2	PT41 (2)	CC97
16	G	2004	11	PT7 (10), PT41 (1)	CC97, CC126
17	H	2003	30	[PT25 (2), **PT26 (17)**, PT27 (5)], PT28 (1), [PT31 (1), PT35 (1), PT36 (1)], PT39 (1), PT51 (1)	CC1, CC97, CC126
18	I	2004	41	[PT7 (1), **PT11 (21)**, PT18 (3), PT19 (3)], PT21 (2), PT31 (6), [PT41 (1), PT42 (2)], PT48 (2)	CC97, CC126, CC133

*Year in which milk samples were collected in each herd.
†PTs listed in square brackets were considered to be closely related as they differed by no more than three bands from the most commonly occurring PT (shown in bold).
‡CCs corresponding to the PTs found in each herd.

The predominance of a limited number of closely related PTs responsible for bovine mastitis from distinct herds and within herds suggests that strains belonging to these PTs have a greater capacity to spread and cause intramammary infection. Other studies suggesting that relatively few clones are responsible for the majority of the *S. aureus* mastitis cases in dairy cattle have been reported (Matthews *et al.*, 1994; Kapur *et al.*, 1995; Fitzgerald *et al.*, 1997; Larsen *et al.*, 2000; Mork *et al.*, 2005). However, it is difficult to compare the genotypes identified by the different methods used in these studies and thus to speculate on the spread of the various genotypes around the world.

A group of 62 *S. aureus* isolates was selected for MLST analysis, including at least one strain of each PT. Isolates recovered from animals in distinct herds and collected in different years were included in this group. A total of 11 STs was detected among the isolates analysed. Seven new alleles and seven new STs were identified and submitted to the *S. aureus* MLST database. The new alleles contained only one nucleotide difference from the alleles already described in the *S. aureus* MLST database. According to the analysis by eBURST v3, the *S. aureus* strains tested in the present study belonged to six CCs when compared with the entire *S. aureus* MLST database (Table 2). A CC is defined as a group of isolates that share at least six of seven alleles with another ST in the group (Feil *et al.*, 2004).

In the present study, PFGE was more discriminatory than MLST. PTs with similarity percentages greater than 65 % belonged to the same CC. Most of the PTs ($n=47$) belonged to CC126 or CC97. PTs belonging to CC126 or CC97 were recovered from 13 herds, in eight municipal districts, and 14 herds, in nine municipal districts, respectively (Table 1, Fig. 2).

Approximately half of the PTs ($n=28$) analysed by MLST, including PT11 and its closely related PTs ($n=16$), belonged to CC126. These PTs (PT1–PT28) had similarity percentages ranging from 68 to 96 % and up to five band differences compared with PT11. Nineteen PTs (PT29–PT47), with similarity percentages ranging from 66 to 96 %, belonged to CC97. PT31 and its closely related PTs ($n=6$) were assigned to this CC. Of 18 PTs, 11 had up to six band differences from PT31. Seven PTs (PT49–PT55), with similarity percentages ranging from 74 to 92 %, belonged to CC1. Between two and eight band differences were observed among these PTs. They were found in four

Table 2. Distribution of STs among CCs found in cows with mastitis in herds from Rio de Janeiro state, Brazil

The allelic profile of each ST is also shown.

CC	ST	Allelic profile						
		arcC	*aroE*	*glpF*	*gmk*	*pta*	*tpi*	*yqiL*
CC1	ST1	1	1	1	1	1	1	1
	ST743*	1	130†	1	1	1	1	1
CC5	ST744*	1	4	1	72†	12	1	10
CC30	ST30	2	2	2	2	6	3	2
CC97	ST97	3	1	1	1	1	5	3
	ST742*	3	131†	1	1	1	5	3
	ST746*	3	1	1	1	1	5	92†
	ST747*	3	1	1	1	1	5	96†
CC126‡	ST126	3	68	1	4	1	5	40
	ST741*	88†	68	1	4	1	5	40
CC133	ST745*	6	66	46	2	99†	50	18

*Novel STs.

†Novel alleles.

‡The group including ST126 and ST741 was named after CC126 although the primary founder of this group cannot be predicted to date.

herds located in four municipal districts. Three PTs (PT58, PT59 and PT60) belonged to CC30, with similarity percentages ranging from 44 to 82 %. PT58 and PT59 differed by three bands, whilst PT60 differed by more than six bands from PT58 and PT59. PTs belonging to CC30 were found in four herds located in four municipal districts. Two PTs (PT56 and PT57), isolated from the same herd, belonged to CC5. They had a similarity of 94 % and only one band difference between them. Only one PT (PT48), found in two herds located in different municipal districts, belonged to CC133 (Table 1, Fig. 2).

Some PTs were assigned to the same CC but had more than four or sometimes more than six band differences. Most of these PTs were found within the same herd, suggesting that they had a common ancestor. Mastitis caused by *S. aureus* is a chronic infection, which may persist for several months (Smith *et al.*, 2005b) and may allow evolution of the PTs by mutation.

Although few studies have analysed bovine *S. aureus* isolates by MLST, all CCs detected in the Brazilian herds have also been found in *S. aureus* isolates from mastitis cases in other countries (Smith *et al.*, 2005a). In our study, the majority of *S. aureus* isolates were identified as members of CC126 and CC97. CC97 was recently described in one study that analysed bovine *S. aureus* isolates from Chile, the USA and the UK (Smith *et al.*, 2005a). Bovine isolates of CC97 were the most frequently detected in Chile (milk samples) and in the USA (mainly milk samples), but they were not detected in the UK. Strains of ST126 were also detected in the same study but only in herds in the USA (milk and milking machine liner samples) (Smith *et al.*, 2005a). CC97 was also identified in another study including a large number of *S. aureus* isolates from the UK, but all were recovered from cows and the environment of a single organic dairy farm (Smith *et al.*, 2005b). CC97 was also infrequent among isolates recovered from bovine bulk milk and raw-milk products in Norway (Jorgensen *et al.*, 2005).

The second most prevalent CC found in this study (CC97) has also been detected among *S. aureus* isolates from the majority of bovine mastitis cases investigated in other countries located in different continents. Isolates belonging to CC126 were the most frequently recovered group in this study, showing that different CCs may predominate in distinct geographical areas. Recently, Aires-de-Sousa *et al.* (2007) reported the isolation of these two CCs from bovine mastitis in another region of Rio de Janeiro state. However, isolates belonging to CC97 were predominant. In contrast to CC126, CC97 was also recovered from other ruminants (goat, sheep and buffalo) with mastitis.

CC97 and CC126 have been rarely and never detected among isolates from human beings, respectively (Feil *et al.*, 2003; Smith *et al.*, 2005a; www.mlst.net). These observations suggest that these strains are specific for ruminant intramammary infections. Nevertheless, important CCs associated with *S. aureus* infection in human beings (CC1, CC5 and CC30) worldwide, including Brazil, were also found in isolates from cows in six herds located in five municipal districts. Isolates belonging to CCs associated with infections in human beings have also been observed in other studies from bovine isolates (Smith *et al.*, 2005a, b), including meticillin-resistant *S. aureus* (MRSA) strains (Kwon *et al.*, 2005). This finding indicates the possibility of transmission of strains between humans and bovines/dairy products, although it does not seem to be a frequent event (Zadoks *et al.*, 2000; Lee, 2003). Isolates belonging to CC1, CC5 and CC30 have frequently been isolated from asymptomatic carriers or patients with invasive diseases (Enright *et al.*, 2000; Feil *et al.*, 2003; Aguiar-Alves *et al.*, 2006; Layer *et al.*, 2006; Vivoni *et al.*, 2006). Studies have suggested that the genetic backgrounds of these CCs have features allowing enhanced capacity of spread among human beings (Gomes *et al.*, 2006). In addition, CC5 and CC30 correspond to some of the current major epidemic MRSA lineages responsible for hospital-acquired and/or community-acquired MRSA (Ribeiro *et al.*, 2005; Gomes *et al.*, 2006). In the present work, isolates belonging to these CCs were meticillin susceptible (results not shown).

In conclusion, this study evaluated the genetic diversity of *S. aureus* isolates from cows with mastitis in Brazilian herds. The majority of infections were caused by a limited number of clones, which were related to international circulating bovine *S. aureus* lineage strains. In addition, the analysis of isolates from a geographical region that has scarcely been surveyed by MLST identified new STs. The occurrence of widespread bovine mastitis-associated CC *S.*

aureus strains suggests that they carry traits that enhance their capacity to cause infection or survive in the bovine host. Investigation of specific traits of these lineages might provide information about possible targets for mastitis control measures.

ACKNOWLEDGEMENTS

This study was supported by Ministério da Ciência e Tecnologia (MCT/PRONEX), Conselho Nacional de Desenvolvimento Científico e Tecnológico (CNPq), Fundação Carlos Chagas de Amparo a Ciência do Estado do Rio de Janeiro (FAPERJ), Coordenação de Aperfeiçoamento de Pessoal de Nível Superior (CAPES) and the Fogarty International Program in Global Infectious Diseases (TW006563) of the National Institutes of Health.

REFERENCES

Aguiar-Alves, F., Medeiros, F., Fernandes, O., Pereira, R. M. G., Perdreau-Remington, F. & Riley, L. W. (2006). New *Staphylococcus aureus* genotyping method based on exotoxin (*set*) genes. *J Clin Microbiol* **44**, 2728–2732.

Aires-de-Sousa, M., Parente, C. E. S. R., Vieira-da-Motta, O., Bonna, I. C. F., Silva, D. A. & Lencastre, H. (2007). Characterization of *Staphylococcus aureus* isolates from buffalo, bovine, ovine, and caprine milk samples collected in Rio de Janeiro state, Brazil. *Appl Environ Microbiol* **73**, 3845–3849.

Brito, M. A. V. P., Brito, J. R. F., Ribeiro, M. T. & Veiga, V. M. O. (1999). Padrão de infecção de intramamária em rebanhos leiteiros: exame de todos os quartos mamários das vacas em lactação. *Arq Bras Med Vet Zootec* **51**, 129–135.(in Brazilian)

Enright, M. C. & Spratt, B. G. (1999). Multilocus sequence typing. *Trends Microbiol* **7**, 482–487.

Enright, M. C., Day, N. P. J., Davies, C. E., Peacock, S. J. & Spratt, B. G. (2000). Multilocus sequence typing for characterization of methicillin-resistant and methicillin-susceptible clones of *Staphylococcus aureus*. *J Clin Microbiol* **38**, 1008–1015.

Enright, M. C., Robinson, D. A., Randle, G., Feil, E. J., Grundmann, H. & Spratt, B. G. (2002). The evolutionary history of methicillin-resistant *Staphylococcus aureus* (MRSA). *Proc Natl Acad Sci U S A* **99**, 7687–7692.

Feil, E. J., Copper, J. E., Grundmann, H., Robinson, D. A., Enright, M. C., Berendt, T., Peacock, S. J., Smith, J. M., Murphy, M. & other authors (2003). How clonal is *Staphylococcus aureus*? *J Bacteriol* **185**, 3307–3316.

Feil, E. J., Li, B. C., Aanensen, D. M., Hanage, W. P. & Spratt, B. G. (2004). eBURST: inferring patterns of evolutionary descent among clusters of related bacterial genotypes from multilocus sequence typing data. *J Bacteriol* **186**, 1518–1530.

Fitzgerald, J. R., Meaney, W. J., Hartigan, P. J., Smyth, C. J. & Kapur, V. (1997). Fine-structure molecular epidemiological analysis of *Staphylococcus aureus* recovered from cows. *Epidemiol Infect* **119**, 261–269.

Gomes, A. R., Westh, H. & de Lencastre, H. (2006). Origins and evolution of methicillin-resistant *Staphylococcus aureus* clonal lineages. *Antimicrob Agents Chemother* **50**, 3237–3244.

Joo, Y. S., Fox, L. K., Davis, W. C., Bohach, G. A. & Park, Y. H. (2001). *Staphylococcus aureus* associated with mammary glands of cows: genotyping to distinguish different strains among herds. *Vet Microbiol* **80**, 131–138.

Jorgensen, H. J., Mork, T., Caugant, D. A., Kearns, A. & Rorvik, L. M. (2005). Genetic variation among *Staphylococcus aureus* strains from Norwegian bulk milk. *Appl Environ Microbiol* **71**, 8352–8361.

Kapur, V., Sischo, W. M., Greer, R. S., Whittman, T. S. & Musser, J. M. (1995). Molecular population genetic analysis of *Staphylococcus aureus* recovered from cows. *J Clin Microbiol* **33**, 376–380.

Kwon, N. H., Park, K. T., Moon, J. S., Jung, W. K., Kim, S. H., Kim, J. M., Hong, S. K., Koo, H. C., Joo, Y. S. & Park, Y. O. (2005). Staphylococcal cassette chromosome *mec* (SCC*mec*) characterization and molecular analysis for methicillin-resistant *Staphylococcus aureus* and novel SCC*mec* subtype IVg isolated from bovine milk in Korea. *J Antimicrob Chemother* **56**, 624–632.

Larsen, H. D., Sloth, K. H., Elsberg, C., Enevoldsen, C., Pedersen, L. H., Eriksen, N. H., Aarestrup, F. M. & Jensen, N. E. (2000). The dynamics of *Staphylococcus aureus* intramammary infection in nine Danish dairy herds. *Vet Microbiol* **71**, 89–101.

Layer, F., Ghebremedhin, B., Konig, W. & Konig, B. (2006). Heterogeneity of methicillin-susceptible *Staphylococcus aureus* strains at a German University Hospital implicates the circulating-strain pool as a potential source of emerging methicillin-resistant *S. aureus* clones. *J Clin Microbiol* **44**, 2179–2185.

Lee, J. H. (2003). Methicillin (oxacillin)-resistant *Staphylococcus aureus* strains isolated from major food animals and their potential transmission to humans. *Appl Environ Microbiol* **69**, 6489–6494.

Lovseth, A., Loncarevic, S. & Berdal, K. G. (2004). Modified multiplex PCR method for detection of pyrogenic exotoxin genes in staphylococcal isolates. *J Clin Microbiol* **42**, 3869–3872.

Matthews, K. R., Kumar, S. J., O'Conner, S. A., Harmon, R. J., Pankey, J. W., Fox, L. K. & Oliver, S. P. (1994). Genomic fingerprints of *Staphylococcus aureus* of bovine origin by polymerase chain reaction-based DNA fingerprinting. *Epidemiol Infect* **112**, 177–186.

Mork, T., Tollersrud, T., Kvitle, B., Jorgensen, H. J. & Waage, S. (2005). Comparison of *Staphylococcus aureus* genotypes recovered from cases of bovine, ovine and caprine mastitis. *J Clin Microbiol* **43**, 3979–3984.

National Mastitis Council (1999). *Laboratory Handbook on Bovine Mastitis*. Madison, WI: National Mastitis Council.

Rabello, R. F., Souza, C. R., Duarte, R. S., Lopes, R. M., Teixeira, L. M. & Castro, A. C. (2005). Characterization of *Staphylococcus aureus* isolates recovered from bovine mastitis in Rio de Janeiro, Brazil. *J Dairy Sci* **88**, 3211–3219.

Ribeiro, A., Dias, C., Silva-Carvalho, M. C., Berquó, L., Ferreira, F. A., Santos, R. N. S., Ferreira-Carvalho, B. T. & Figueiredo, A. M. (2005). First report of infection with community-acquired methicillin-resistant *Staphylococcus aureus* in South America. *J Clin Microbiol* **43**, 1985–1988.

Robinson, D. A. & Enright, M. C. (2004). Multilocus sequence typing and the evolution of methicillin-resistant *Staphylococcus aureus*. *Clin Microbiol Infect* **10**, 92–97.

Smith, E. M., Green, L. E., Medley, G. F., Bird, H. E., Fox, L. K., Schukken, Y. H., Kruze, J. V., Bradley, A. J., Zadoks, R. N. & Dowson, C. G. (2005a). Multilocus sequence typing of intercontinental bovine *Staphylococcus aureus* isolates. *J Clin Microbiol* **43**, 4737–4743.

Smith, E. M., Green, L. E., Medley, G. F., Bird, H. E. & Dowson, C. G. (2005b). Multilocus sequence typing of *Staphylococcus aureus* isolated from high-somatic-cell-count cows and the environment of an organic dairy farm in the United Kingdom. *J Clin Microbiol* **43**, 4731–4736.

Tenover, F. C., Arbeit, R. D., Goering, R. V., Mickelsen, P. A., Murray, B. E., Persing, D. H. & Swaminathan, B. (1995). Interpreting chromosomal DNA restriction patterns produced by pulsed-field gel electrophoresis: criteria for bacterial strain typing. *J Clin Microbiol* **33**, 2233–2239.

Vivoni, A. M., Diep, B. A., Magalhães, A. C. G., Santos, K. R. N., Riley, L. W., Sensabaugh, G. F. & Moreira, B. M. (2006). Clonal composition of *Staphylococcus aureus* isolates at a Brazilian university hospital: identification of international circulating lineages. *J Clin Microbiol* **44**, 1686–1691.

Wilson, D. J., Gonzalez, R. N. & Das, H. H. (1997). Bovine mastitis pathogens in New York and Pennsylvania: prevalence and effects on somatic cell count and milk production. *J Dairy Sci* **80**, 2592–2598.

Zadoks, R., van Leeuwen, W., Barkema, H., Sampimon, O., Verbrugh, H., Schukken, Y. H. & van Belkum, A. (2000). Application of pulsed-field gel electrophoresis and binary typing as tools in veterinary clinical microbiology and molecular epidemiologic analysis of bovine and human *Staphylococcus aureus* isolates. *J Clin Microbiol* **38**, 1931–1939.
